# Effects of Acute Exercise and Carbohydrate Intake on Plasma GDF-15 Levels and Its Association With Appetite Regulation

**DOI:** 10.1210/jendso/bvaf013

**Published:** 2025-01-18

**Authors:** Ellen James, James Frampton, Kevin G Murphy, Edward S Chambers

**Affiliations:** Department of Metabolism, Digestion and Reproduction, Faculty of Medicine, Imperial College London, London W12 0NN, UK; Department of Metabolism, Digestion and Reproduction, Faculty of Medicine, Imperial College London, London W12 0NN, UK; Department of Metabolism, Digestion and Reproduction, Faculty of Medicine, Imperial College London, London W12 0NN, UK; Department of Metabolism, Digestion and Reproduction, Faculty of Medicine, Imperial College London, London W12 0NN, UK

**Keywords:** appetite, metabolism, gastrointestinal hormones, exercise

## Abstract

**Background:**

Growth differentiation factor 15 (GDF-15) is a potential therapeutic target for obesity due to its role in appetite suppression. Although acute exercise stimulates GDF-15 secretion, its relationship with appetite regulation remains unclear. It is also unknown whether preexercise carbohydrate intake would affect GDF-15 responses. This study aimed to examine the effects of acute exercise and carbohydrate intake on GDF-15 secretion and its potential links to appetite regulation.

**Methods:**

In a secondary analysis of a randomized crossover trial, 12 healthy males completed four 120-minute trial conditions: (1) control (water) with rest, (2) control with exercise (0-30 minutes at ∼75% of maximal oxygen uptake), (3) carbohydrate (75 g maltodextrin) with rest, and (4) carbohydrate with exercise. Plasma GDF-15 levels were measured at 0, 30, 60, and 120 minutes, alongside subjective appetite ratings using visual analog scales. Energy intake was measured at the end of each trial condition with an ad libitum meal.

**Results:**

Time-averaged area under the curve analysis showed that neither exercise [34 pg/mL (95% confidence interval [CI], −2-69 pg/mL); *P* = .062) nor carbohydrate intake [10 pg/mL (95% CI, −39-58 pg/mL); *P* = .673] independently or interactively (*P* = .283) affected GDF-15 levels. Exercise induced a delayed independent increase in GDF-15 at 120 minutes [55 pg/mL (95% CI, 18-94 pg/mL); *P* = .008]. No significant associations were found between GDF-15 levels and subjective appetite ratings or energy intake.

**Conclusion:**

A 30-minute bout of high-intensity exercise induces a delayed increase in GDF-15 levels, which is not affected by carbohydrate intake. Physiological GDF-15 responses to acute exercise display no association with markers of appetite regulation.

Growth differentiation factor 15 (GDF-15) is a cytokine in the transforming growth factor β family that has attracted attention as a potential therapeutic target for obesity and its associated metabolic conditions [1]. The release of GDF15 is triggered by various physiological and pathological factors, including cellular and metabolic stress [1]. Exercise, as an acute metabolic stressor, impacts multiple metabolic processes partly through the secretion of signaling molecules from various organs and tissues, collectively defined as exerkines [2]. Research has identified GDF-15 as an exerkine, released from the liver [3] and skeletal muscle [4] in response to acute exercise. Previous studies have shown this effect predominantly with prolonged exercise (> 60 minutes) [3-5] performed by experienced recreational athletes [6-8]. However, it remains uncertain whether shorter bouts of exercise (∼30 minutes), as recommended by health guidelines [9], affect GDF-15 levels in untrained individuals. Understanding this could elucidate the potential role of GDF-15 as an exerkine that contributes to the health benefits of regular exercise.

The relationship between exercise and appetite, known as exercise-induced anorexia, is well established and has been primarily attributed to transient increases in anorexigenic [glucagon-like peptide 1 (GLP-1) and peptide YY (PYY)] and reductions in orexigenic (ghrelin) gastrointestinal hormones [10, 11]. GDF-15 has also been linked to appetite regulation; studies in mice show that acute systemic GDF-15 administration reduces body weight and food intake [12]. These appetite-suppressing effects are mediated through stimulation of its receptor, GDNF family receptor alpha-like, in appetite-regulatory regions of the hindbrain [13]. Therefore, it is postulated that exercise-induced elevations in GDF-15 also contribute to appetite suppression [14]. However, research exploring the associations between exercise, GDF-15 levels, and appetite markers is currently sparse [15].

Previous investigations examining the impact of acute exercise on GDF-15 have been conducted in either overnight-fasted [3, 4] or postprandial conditions [5, 15], and no study has compared this response in different prandial states. Metabolic responses to acute exercise are modulated by preexercise feeding, including carbohydrate intake [16]. While acute carbohydrate ingestion does not significantly affect circulating GDF-15 levels at rest [17], it is unknown if exogenous carbohydrate would impact the GDF-15 response to acute exercise.

This project therefore aimed to investigate the independent and interactive effects of acute exercise and carbohydrate intake on GDF-15 excretion in untrained adults and the association with appetite responses. We hypothesized that acute exercise would independently promote GDF-15 release and that elevated plasma GDF-15 levels would be associated with reduced appetite responses.

## Methods

This is a secondary analysis of a previously published trial [18] that investigated the impact of acute carbohydrate intake and aerobic exercise on plasma hormonal and metabolic responses. The study was approved by South West-Frenchay Research Ethics Committee (19/SW/007) and is registered at clinicaltrials.gov (NCT04019418). All procedures were conducted in line with the Declaration of Helsinki, and participants provided informed consent before enrollment.

### Participants

This study recruited 12 healthy male participants aged 18 to 40 years old, with a body mass index of 18 to 30 kg/m^2^ (inclusive) between February 2019 and February 2020. Participants had an age of 24 ± 5 years (mean ± SD), with a body mass index of 21.9 ± 2.1 kg/m^2^ and a maximal oxygen uptake (VO_2max_) of 40.2 ± 8.7 mL/kg/min. All participants had a VO_max_ <55 mL/kg/min.

### Study Design and Interventions

The study design has been described in detail elsewhere [18]. In brief, this study employed a semidouble-blind, randomized, 4-period crossover, placebo-controlled design. Initially, participants attended a screening visit for eligibility assessment. Eligible participants returned for a VO_2max_ test to determine absolute exercise intensity for subsequent visits. Each participant then completed 4 randomized trial conditions: (1) control beverage and rest session (CON-REST), (2) control beverage and exercise session (CON-EX), (3) carbohydrate beverage and rest session (CARB-REST), and (4) carbohydrate beverage and exercise session (CARB-EX). Each visit was separated by a minimum of 3 days. Participants were instructed to avoid strenuous exercise, caffeine, and alcohol; to standardize their evening meal the day before each visit; and to fast overnight from 20:00, with water allowed.

Participants arrived at the research facility at 09:00 ± 1 hour, where an intravenous cannula was inserted for serial blood sampling. After baseline measurements, participants consumed either a control (300 mL water) or carbohydrate (300 mL water and 75 g maltodextrin) beverage within 5 minutes, and then either rested or exercised (continuous 75% VO_2max_) for 30 minutes. Following this, they rested for 90 minutes before being provided an ad libitum meal. Venous blood samples and 100 mm visual analog scale scores to assess subjective appetite and nausea were collected throughout.

### Hormone and Metabolite Analysis

GDF-15 levels were assessed in baseline, 30-, 60-, and 120-minute heparin plasma samples using the Quantikine ELISA Human GDF15 Immunoassay (R&D Systems Cat# DGD150, RRID:AB_2877710), following the manufacturer's protocol. All samples were diluted 1:3 and measured in duplicate, with a mean ± SD coefficient of variation of 4.7 ± 4.4% between duplicates. Details on the analysis of other hormones and metabolites have been previously reported [18].

### Statistical Analysis

The time-averaged area under the curve (AUC) was calculated for GDF-15 in each trial condition. A robust linear mixed effects model was then used (R package “robustlmm’ [19]) to evaluate the independent and interactive effects of exercise and carbohydrate on GDF-15 levels. Fixed effects included in the model were exercise and carbohydrate and an interaction between exercise and carbohydrate. Random effects included in the model were participant, an interaction between participant and exercise, and an interaction between participant and carbohydrate. Random effects with zero variance were removed from the model. The Satterthwaite's degrees of freedom method implemented in the R package “lmerTest” [20] was used to derive *P*-values for fixed effects. If a significant interaction effect between exercise and carbohydrate was detected, multiple comparisons of estimated marginal means were performed using the R package “emmeans” [21], with the Tukey adjustment being applied. Estimated marginal means of exercise and carbohydrate were calculated when no significant interaction effect was detected. Statistical significance was set at *P* < .05.

Repeated measures correlation (Rmcorr) analyses [22, 23] were conducted between the time-averaged AUC of GDF-15 and unadjusted values for ad libitum energy intake and energy balance (total energy intake minus total energy expenditure) in each trial condition. These analyses would identify if common within-participant linear associations exist between plasma GDF-15 levels and energy intake and energy balance. Rmcorr analyses were then used on GDF-15 levels and those of subjective appetite and nausea visual analog scale scores and other hormones (GLP-1, PYY, ghrelin, insulin, and glucagon) measured at 0, 30, 60, and 120 minutes. These analyses would identify if common within-participant linear associations exist between the temporal changes in GDF-15 and subjective appetite and hormone responses in each trial condition. *P*-values from Rmcorr were corrected for false discovery rate (Q) = 5% [24]. Statistical significance was set at *P* < .05.

## Results

### Independent and Interactive Effects of Acute Exercise and Carbohydrate Intake on GDF-15

**Figure 1.**
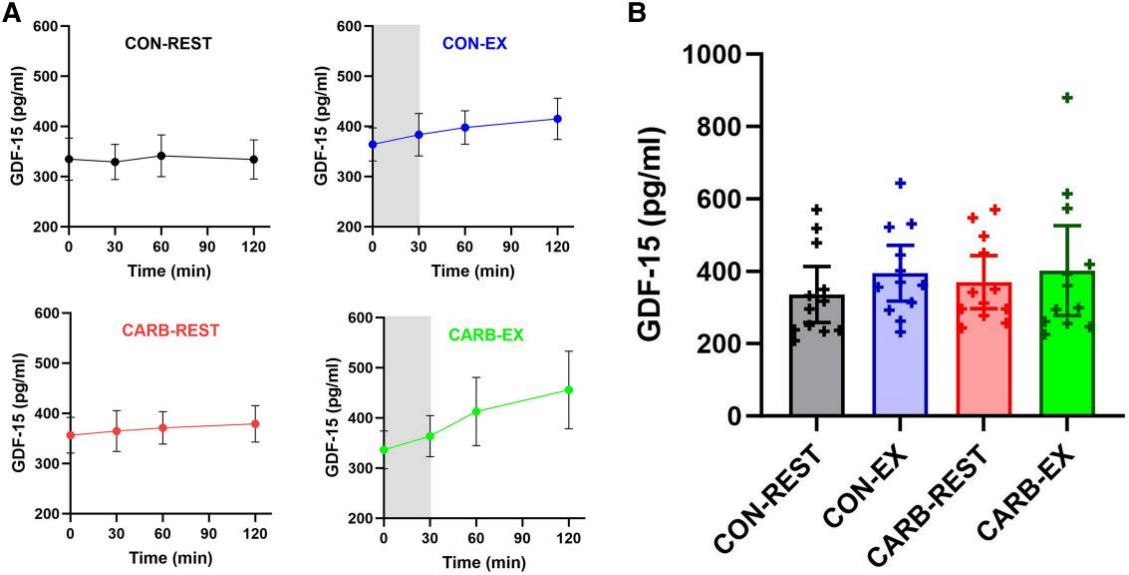
(A) Temporal responses for GDF-15 in the CON-REST, CON-EX, CARB-REST) and CARB-EX trial conditions (mean ± SEM; n = 12). Shaded area indicates the time that aerobic exercise (∼75% VO_2max_) was completed in the CON-EX and CON-REST trial conditions. (B) Time-averaged area under the curve for GDF-15. Crosses represent values for individual participants (Mean ± SEM; n = 12). Abbreviations: CARB-EX, Carbohydrate-Exercise; CARB-REST, Carbohydrate-Exercise; CON-EX, Control-Exercise; CON-REST, Control-Rest; GDF-15, growth differentiation factor 15.

The temporal response of GDF-15 in each trial condition and the time-averaged AUC are shown in Fig. 1A and B. Analyses using AUC data revealed that exercise [34 pg/mL (95% CI, −2-69 pg/mL); *P* = .062] and carbohydrate [10 pg/mL (95% CI, −39-58 pg/mL); *P* = .673] had no independent or interactive (*P* = .283) effects on GDF-15. As GDF-15 levels peaked at the end of the exercise conditions (Fig. 1A), further exploratory analyses were conducted using the 120-minute data in the same robust linear mixed effects model. This identified a significant independent effect of exercise [55 pg/mL (95% CI, 18-94 pg/mL); *P* = .008] at the end of the study period, with no independent effect of carbohydrate [23 pg/mL (95% CI, −42-87 pg/mL); *P* = .455] or interaction (*P* = .237).

### GDF-15 Levels and Acute Appetite Regulation

**Figure 2.**
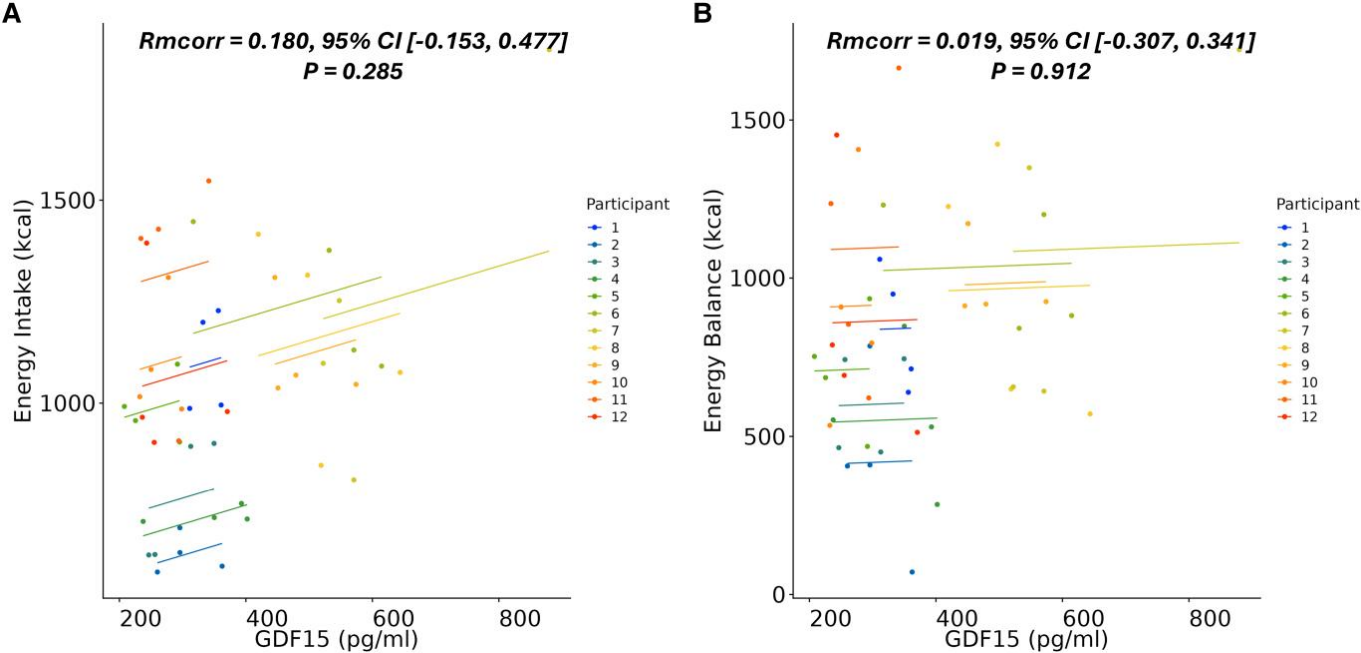
Within-participant Rmcorr between time-averaged area under the curve GDF-15 levels and (A) energy intake and (B) energy balance in each trial condition. *P*-values from Rmcorr were corrected for false discovery rate (Q) = 5% (n = 12). Abbreviations: GDF-15, growth differentiation factor 15; Rmcorr, repeated measures correlations.

Rmcorr analyses did not find an association between GDF-15 levels and ad libitum energy intake or energy balance (Fig. 2) or temporal relationships between GDF-15 responses and subjective appetite and nausea in any of the 4 trial conditions (Table 1). There were no temporal relationships between GDF-15 responses and GLP-1, PYY, ghrelin, glucagon, or insulin in any of the 4 trial conditions (Table 1).

**Table 1.** Rmcorr between GDF-15 levels measured at 0, 30, 60, and 120 minutes in each trial condition and the CAS and nausea visual analog scale scores and GLP-1, PYY, ghrelin, insulin, and glucagon levels measured

	CON-REST		CON-EX		CARB-REST		CARB-EX	
Subjective appetite	Rmcorr	*P*	Rmcorr	*P*	Rmcorr	*P*	Rmcorr	*P*
CAS	−0.235, 95% CI [−.519, 0.097]	.709	0.139, 95% CI [−0.193, .443]	.969	−0.115, 95% CI [−0.423, .217]	.704	0.339, 95% CI [0.017, .598]	.283
Nausea	0.130, 95% CI [−0.203, .436]	.785	−0.063, 95% CI [−0.38, .266]	1.000	0.237, 95% CI [−0.094, .521]	.372	0.000, 95% CI [−0.324, .324]	1.000
**Hormones**								
GLP-1	−0.079, 95% CI [−0.393, .252]	.795	−0.002, 95% CI [−0.326, .322]	1.000	0.120, 95% CI [−0.212, .427]	.704	−0.008, 95% CI [−0.331, .317]	1.000
PYY	−0.046, 95% CI [−0.365, .282]	.795	0.018, 95% CI [−0.308, .34]	1.000	0.264, 95% CI [−0.065, .542]	.372	−0.113, 95% CI [−0.422, .219]	1.000
Ghrelin	0.053, 95% CI [−0.276, .371]	.795	−0.167, 95% CI [−0.466, .166]	.969	−0.238, 95% CI [−0.522, .093]	.372	−0.022, 95% CI [−0.344, .304]	1.000
Insulin	−0.177, 95% CI [−0.474, .156]	.709	−0.071, 95% CI [−0.386, .259]	1.000	0.002, 95% CI [−0.322, .326]	1.000	0.156, 95% CI [−0.177, .457]	1.000
Glucagon	−0.175, 95% CI [−0.472, .158]	.709	0.192, 95% CI [−0.14, .486]	.969	0.075, 95% CI [−0.255, .389]	.778	0.081, 95% CI [−0.249, .395]	1.000

at the same time points. CON-REST, CON-EX, CARB-REST, and CARB-EX. *P*-values from Rmcorr were corrected for false discovery rate (Q) = 5% (n = 12).

Abbreviations: CARB-EX, Carbohydrate-Exercise; CARB-REST, Carbohydrate-Exercise; CAS, composite appetite score; CI, confidence interval; CON-EX, Control-Exercise; CON-REST, Control-Rest; GDF-15, growth differentiation factor 15; GLP-1, glucagon-like peptide 1; PYY, peptide YY; Rmcorr, repeated measures correlations.

## Discussion

This secondary analysis of a randomized crossover study [18] explored the independent and interactive effects of exercise and carbohydrate intake on GDF-15 excretion and examined whether GDF-15 showed consistent within-participant linear associations with markers of appetite regulation.

Our statistical analysis, using the time-averaged AUC over each 120-minute trial condition, revealed that neither acute exercise nor carbohydrate ingestion modulate GDF-15 excretion in healthy untrained males. However, GDF-15 levels peaked in both exercise conditions at the end of the study period, and further exploratory analysis using this final 120-minute data identified delayed effects of acute exercise on GDF-15 release that was independent of carbohydrate intake. Therefore, while the 30-minute high-intensity exercise bout and 90-minute recovery did not significantly increase GDF-15 levels overall, extending the observation period beyond 120 minutes could have potentially revealed a more pronounced rise in GDF-15. This aligns with previous research reporting that GDF-15 levels continue to rise in the hours following exercise compared to those measured in the immediate postexercise period [3, 5].

The 55 pg/mL independent effect of exercise at 120 minutes is considerably lower than some previous studies reporting >500 pg/mL elevations in the postexercise period [6-8]. However, these studies typically employed prolonged bouts of exercise (>120 minutes) in experienced recreational athletes that are not representative of the shorter accumulated bouts of exercise recommended for health benefits in the general population [9]. A limitation of previous studies examining the impact of acute exercise on GDF-15 release is the absence of a time-matched, resting control arm [3, 4, 7, 8]; thus exercise-induced changes cannot be distinguished from possible diurnal secretory responses [25]. The lower exercise-induced GDF-15 secretion in the current study may therefore be partly explained by the comparison with appropriate resting control conditions.

Despite the proposed role of GDF-15 in appetite regulation, we did not identify that trial conditions with higher GDF-15 levels were commonly associated with lower ad libitum energy intake or total energy balance. Temporal analysis in each study condition also found no significant association between GDF-15 responses and subjective appetite or nausea. This outcome aligns with a recent investigation reporting that acute exercise in adults with overweight and obesity was not associated with markers of appetite regulation [15]. The relatively small physiological changes in GDF-15 in the current study may not have been sufficient to impact appetite responses. Indeed, rodent studies show that physiological levels of GDF-15 after exercise do not replicate the pharmacological effects of GDF-15 on appetite [6]. A limitation of the current study is that ad libitum energy intake was assessed only once, 90 minutes after the cessation of exercise. Given the observation that GDF-15 levels may have continued to rise with further postexercise blood samples, future studies should examine postexercise appetite responses and energy intake over an extended 24-hour period.

Our temporal analysis did not reveal consistent within-participant linear relationships between GDF-15 and other appetite-regulating hormones, such as ghrelin, GLP-1, and PYY. In contrast to GDF-15, which continues to increase in the hours following exercise [3, 5], the response of ghrelin, GLP-1, and PYY peak during or immediately after exercise and quickly return to preexercise concentrations [10, 11, 18]. Additionally, while carbohydrate intake influences the secretion of ghrelin, GLP-1, and PYY [18], our study confirms previous findings that GDF-15 is unaffected by acute carbohydrate feeding [17]. This suggests that the mechanisms driving exercise-induced GDF-15 secretion differ from those regulating other exercise-induced hormonal responses. Although prior studies have suggested that glucagon and insulin may regulate GDF-15 release [3], we found no association between the temporal responses of these glucose-regulatory hormones and GDF-15 levels under any trial condition.

Several important limitations should be considered. Our participants were exclusively young (<40 years) healthy males, limiting the wider applicability of our findings. Previous research has reported age and sex differences [26] and elevated levels of GDF-15 in obese individuals and those with type-2 diabetes [27], underscoring the need for caution when extrapolating our findings. The specific carbohydrate dose and exercise intensity were selected based on their known ability to modulate gastrointestinal hormone release [28, 29]; different combinations of macronutrients and exercise types could therefore yield distinct independent and interactive effects on GDF-15 release.

In conclusion, our study does not support a role for GDF-15 as an exerkine that modulates acute appetite regulation following 30 minutes of continuous high-intensity exercise in untrained males. Instead, our findings support a distinct postexercise secretory profile for GDF-15 that differs from other appetite-regulatory hormones. The significant independent effect of exercise at the 120-minute time point highlights the importance of extended postexercise observation periods when examining the potential role of GDF-15 in postexercise metabolism. These findings provide important information for comprehensive investigations into the complex interplay between exercise, nutrition, and GDF-15 in metabolic health.

## Data Availability

Raw data used for analysis are available from the Open Science Framework at: https://osf.io/pe5um/

## Clinical Trials Registration

Registered at clinicaltrials.gov (NCT04019418).
